# Inflammatory myofibroblastic tumor of the ileocecal area complicated by intussusception: a case report in a child

**DOI:** 10.3389/fped.2025.1569750

**Published:** 2025-04-08

**Authors:** Guohou He, Yonghan Luo, Yanchun Wang, Qiang Bai

**Affiliations:** ^1^ Department of General Surgery, Kunming Children’s Hospital, Kunming, China; ^2^ Second Department of Infectious Diseases, Kunming Children’s Hospital, Kunming, China; ^3^ School of Life Sciences and Technology, Kunming University of Science and Technology, Kunming, China

**Keywords:** inflammatory myofibroblastic tumor, secondary intussusception, pediatric, surgical procedures, misdiagnosis

## Abstract

Inflammatory myofibroblastic tumor (IMT) is a rare mesenchymal tumor that can occur in multiple organs. This case report presented an unusual instance of ileocecal IMT in a child complicated by secondary intussusception. A 2-year-6-month-old male was admitted with abdominal pain and fever for 15 days, progressing to currant jelly stools for 2 days. Physical examination revealed a right abdominal mass, and emergency laparotomy confirmed ileocolic intussusception with a firm mass in the ileocecal region. Pathological examination confirmed the diagnosis of IMT. IMT as a secondary cause in pediatric ileocecal intussusception is rare when encountering an atypical intussusception with a suspect lead-point, IMT should be considered.

## Introduction

Inflammatory myofibroblastic tumor (IMT) is a rare mesenchymal tumorthat primarily affects children and young adults (1). While IMTs can originate from multiple tissue types, they predominantly develop from soft tissue or visceral organs and exhibit variable radiological and histological features. Clinical outcomes include spontaneous regression, local recurrence, and widespread metastasis. IMTs are most located in the lungs, with the most frequently reported extrathoracic sites being the mesentery and retroperitoneum (2, 3). Previous reports (4–6) of IMT have primarily focused on adults. In children, abdominal IMTs have been reported in the stomach, liver, spleen, and pancreas; however, those occurring in the ileocecal region are rare (7–9). Furthermore, the complication of intussusception is exceptionally uncommon. Here, we reported a recent case from our hospital of an ileocecal IMT complicated by cecal intussusception.

## Clinical case

A 2-year-and-6-month-old male child was admitted with a chief complaint of abdominal pain and fever that had persisted for 15 days, worsening with currant jelly stools for 2 days. His symptoms began 15 days before admission with paroxysmal mild periumbilical abdominal pain, accompanied by intermittent fever with a peak of 39.5°C. A diagnosis of “acute mesenteric lymphadenitis” was established at a local community hospital, primarily based on clinical presentation, including fever and abdominal pain. However, no imaging studies or laboratory tests were performed at that time. The patient received anti-inflammatory therapy and intravenous fluids, leading to symptomatic relief. Two days prior to admission, the child experienced a recurrence of paroxysmal abdominal pain, which worsened compared to before. He was subsequently diagnosed with “intussusception” at another facility, a local county hospital, where abdominal ultrasonography revealed the characteristic “target sign.” He underwent an unsuccessful barium enema reduction. Following the second episode of paroxysmal abdominal pain, and just before his transfer to our hospital, he experienced two episodes of currant jelly stool. The child was then referred to our hospital for further evaluation and admission.

Physical examination: A sausage-shaped mass was palpable in the right mid-abdomen, firm in consistency, with clear borders, relatively fixed, and tender to palpation. The mass was first detected upon the child's presentation at our hospital, not at the initial visit.

Laboratory and imaging examination: White blood cell count: 6.4 × 10^9^/L (reference range: 4.8-14.6 × 10^9^/L);C-reactive protein (CRP): 198.56 mg/L (reference range: 0–10 mg/L); Liver and kidney function tests, coagulation parameters, and blood cultures were all negative.

**Figure 1:**
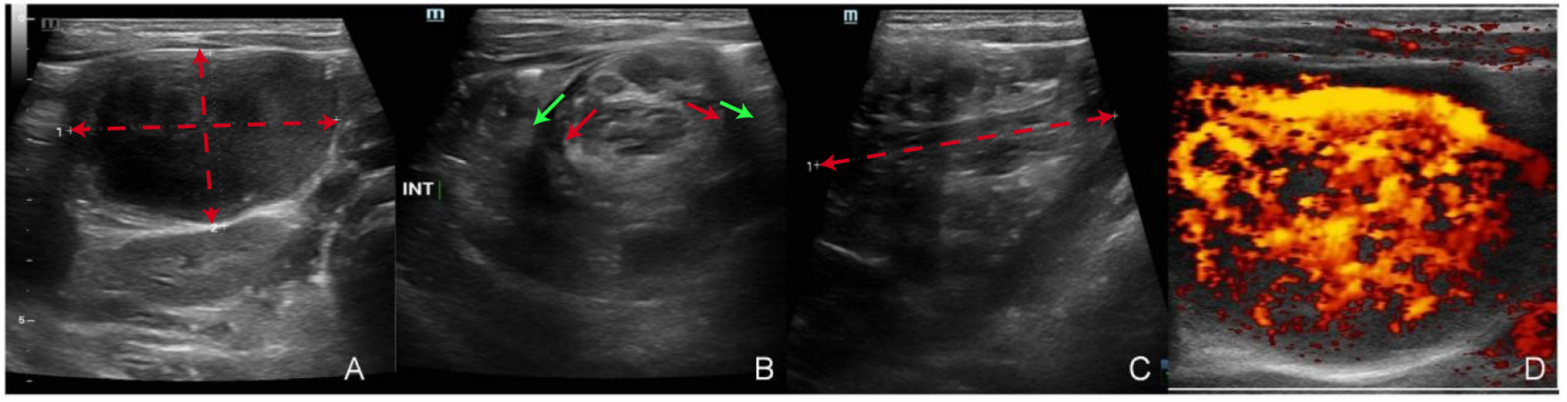
Abdominal ultrasound findings. **(A)** Hypoechoic mass detected in the abdominal cavity, with red dashed lines outlining its dimensions (5.7 × 3.3 × 2.8 cm). **(B)** Intussusception displaying distinct inner and outer layers of the intestinal wall, highlighted by red arrows (inner layers) and green arrows (outer layers). **(C)** Longitudinal view of the intussusception, with the red dashed line indicating the longitudinal axis. **(D)** Doppler imaging revealing a hypervascular mass with abundant blood supply.

Abdominal ultrasound (see Figure 1): A mass was detected in the right upper abdomen, measuring approximately 5.7 × 3.3 × 2.8 cm. The colon transverse section showed a “target sign,” and the longitudinal section demonstrated a “cuff” sign. No peristalsis was observed dynamically. Several mesenteric lymph nodes were visible within the mass, with the largest measuring approximately 0.8 × 0.5 cm. A second mass was identified near the primary mass within the ileocecal region, measuring approximately 3.7 × 3.6 × 2.9 cm, with clear borders and low echogenicity. The distinction between the cortex and medulla was unclear.

**Figure 2:**
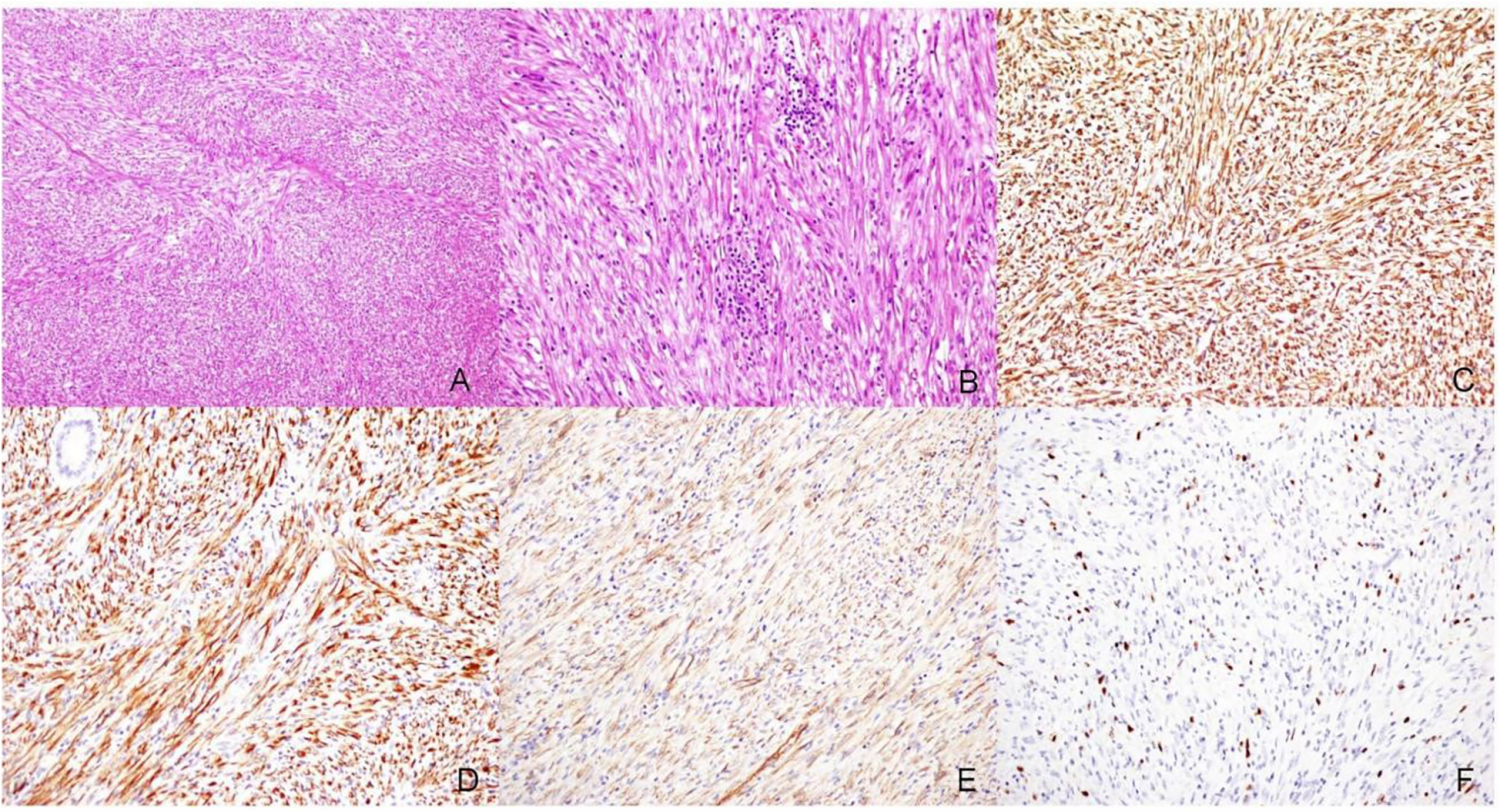
Pathological findings of the surgical specimen. **(A)** Tumor cells are spindle-shaped, short spindle-shaped, or round, arranged in bundles or swirl patterns (HE, ×100). **(B)** The stroma is infiltrated with a large number of inflammatory cells (HE, ×200). **(C)** Vimentin (+) (IHC Envision two-step method, ×200). Vimentin is a marker for cells of mesenchymal origin, supporting the idea that the tumor may arise from mesenchymal tissue. **(D)** ALK (+) (IHC Envision two-step method, ×200). ALK positivity is characterized by diffuse cytoplasmic staining, whereas under normal conditions, ALK expression is almost absent. **(E)** β-catenin (+) (IHC Envision two-step method, ×200), β-catenin positivity may indicate activation of the Wnt signaling pathway, which is commonly observed in desmoid-type fibromatosis (also known as desmoid tumors or fibromatosis). **(F)** Ki-67 (10%) (IHC envision two-step method, ×200).

After admission, the patient underwent preoperative examinations, and an emergency laparotomy was performed. During the surgery, ileocolic intussusception was observed, with the ileum approximately 15 cm from the ileocecal valve telescoping into the cecum. A firm mass, approximately 4.5 × 4 × 3 cm in size, was found within the lumen of the ileocecal region, accompanied by significantly enlarged mesenteric lymph nodes. Approximately 15 cm of the distal ileum and ileocecal region (including the mass and appendix) were excised and sent for pathological examination. Histopathological analysis confirmed the diagnosis of an IMT, with clear surgical margins, indicating complete tumor excision. Immunohistochemistry revealed positivity for Anaplastic Lymphoma Kinase (ALK), which is characteristic of IMTs. The postoperative pathological result, as shown in Figure 2, supports this diagnosis. Enlarged mesenteric lymph nodes were identified during surgery and subsequently excised for metastatic evaluation. Postoperative pathological examination confirmed the absence of lymph node metastasis. The patient's postoperative recovery was uneventful, and he was discharged. The patient has been on regular follow-up for one-year post-surgery, undergoing imaging studies like CT scans every six months to monitor for recurrence. So far, no recurrence has been detected.

## Discussion

Pediatric intussusception can be classified into primary and secondary types. Primary intussusception accounts for the majority of cases and typically has no clear cause. However, it may be associated with lymphoid hyperplasia in the intestinal wall, external stimuli (such as infections), and anatomical factors (such as normal variations in intestinal structure, including increased lymphoid tissue in Peyer's patches or a more mobile mesentery). In contrast, secondary intussusception occurs due to a distinct structural abnormality, such as a tumor, polyp, or Meckel's diverticulum, which serves as a pathological lead point. This distinction helps differentiate the underlying mechanisms of primary and secondary intussusception. Secondary intussusception is more common in older children, with over 60% of cases occurring in those aged 5–14 years (10).

IMT is a rare tumor that primarily affects children and young adults. These tumors are typically benign, though they can exhibit aggressive behavior and local recurrence (9, 11, 12). The exact etiology of IMT remains unclear, but some cases are associated with genetic mutations, while others may be triggered by chronic inflammation or infection (13). The lungs are the most common site of IMT, but it can also affect the mesentery, retroperitoneum, and gastrointestinal tract (7, 14). The ileocecal region is a rare site for these tumors in pediatric patients, and intussusception as a complication is even rarer (12, 15). In pediatric patients presenting with symptoms such as abdominal pain and fever, a high index of suspicion should be maintained for a potential underlying pathological mass (16). In this case, the clinical symptoms, imaging findings, and failure of initial treatment raised concern for a broader differential diagnosis, including bowel perforation and sepsis as primary considerations. However, given the worsening symptoms and the overall presentation, the possibility of an underlying mass, such as IMT, was also strongly considered. This suspicion was ultimately confirmed through intraoperative and pathological examination.

Considering the relative rarity of secondary intussusception caused IMT, we recommend the following measures: For all patients with intussusception, especially those with atypical clinical presentations or cases where reduction by rectal enema has failed, it is crucial to perform imaging studies to differentiate between primary and secondary intussusception. This approach aids in the timely identification of underlying pathological causes, such as tumors or inflammation, thereby guiding subsequent treatment decisions. Ultrasound is widely regarded as the preferred diagnostic modality for intussusception due to its non-invasive nature, absence of radiation exposure, ease of use, and high sensitivity and specificity in diagnosis (17). However, in cases where ultrasound findings are inconclusive or when complications such as bowel ischemia or perforation are suspected, additional imaging modalities like computed tomography (CT) or magnetic resonance imaging (MRI) may be considered to provide further anatomical detail and assess for underlying pathology.

In cases of suspected IMT, the necessity of lymph node resection should be carefully evaluated. While routine lymphadenectomy is not always required, significantly enlarged or suspicious lymph nodes should be considered for surgical excision and pathological examination to rule out metastatic involvement. In cases where IMT exhibits aggressive features or recurrence, consultation with an oncologist is crucial. The management strategy may include chemotherapy or targeted therapy in selected cases, particularly when complete surgical resection is not feasible or in cases with high-risk pathological features (14).

The surgical treatment of IMT typically involves complete excision of the tumor. In this case, due to the presence of intussusception, manual reduction was performed first, followed by the excision of the tumor and a portion of the ileum and cecum. Although IMT is usually benign (9, 18), careful follow-up is necessary due to its potential for recurrence or local spread. In this patient, timely diagnosis and surgical intervention led to symptom relief, and the postoperative recovery was good. This case emphasizes the importance of considering IMT in the differential diagnosis of abdominal masses in children, particularly when accompanied by uncommon complications such as intussusception.

## Data Availability

The original contributions presented in the study are included in the article/Supplementary Material, further inquiries can be directed to the corresponding author.
